# Perspective review: lessons from successful clinical trials and real-world studies of systemic therapy for metastatic pheochromocytomas and paragangliomas

**DOI:** 10.1177/17588359241301359

**Published:** 2024-11-21

**Authors:** Camilo Jimenez, Rene Baudrand, Thomas Uslar, Daniel Bulzico

**Affiliations:** Department of Endocrine Neoplasia and Hormonal Disorders, The University of Texas MD Anderson Cancer Center, 1400 Pressler Street, Unit 1461, Houston, TX 77030, USA; Department of Endocrinology, Pontificia Universidad Catolica de Chile, Santiago, Chile; Department of Endocrinology, Pontificia Universidad Catolica de Chile, Santiago, Chile; Department of Nuclear Medicine and Endocrine Oncology, Brazilian National Cancer Institute, Rio de Janeiro, Brazil

**Keywords:** metastatic pheochromocytoma and paraganglioma, radiopharmaceuticals, tyrosine kinase inhibitors

## Abstract

Pheochromocytomas and paragangliomas (PPGLs) are orphan tumors with the potential to spread to distant organs such as the lymph nodes, the skeleton, the lungs, and the liver. These metastatic tumors exhibit high rates of morbidity and mortality due to their frequently large tumor burden, the progression of the disease, and the excessive secretion of catecholamines that lead to cardiovascular disease and gastrointestinal dysmotility. Several molecular drivers responsible for the development of PPGLs have been described over the last 30 years. Although therapeutic options are limited, substantial progress has been made in the recognition of effective systemic therapies for these tumors. Successful clinical trials with radiopharmaceuticals such as high-specific-activity meta-iodobenzylguanidine and tyrosine kinase inhibitors such as cabozantinib and sunitinib have been recently published. This review will discuss the results of these studies and their impact on current clinical practices. In addition, this review will provide valuable information on how to design clinical trials to treat patients with metastatic PPGLs with novel medications.

## Introduction

Pheochromocytomas and paragangliomas (PPGLs) are rare neuroendocrine tumors. An estimated 1500–2000 new cases of PPGL are diagnosed every year in the United States.^1^ Of these, 25% are metastatic (MPPGL).^2^ MPPGLs are associated with elevated rates of morbidity and mortality related to their tumor burden, speed of progression, and excessive secretion of catecholamines, predisposing patients to cardiovascular disease and other endocrine complications.^3^ These metastases mainly affect the lymph nodes (80%), skeleton (72%), and liver and lungs (50%).^4,5^ In up to 50% of patients with MPPGL, metastases are discovered at the time of diagnosis of the primary tumor; in the other 50%, metastases might be discovered years or decades after the initial discovery of the primary tumor.^4^ Subsequently, long-term follow-up after surgery is recommended, especially in individuals with clinical predictors for metastases such as large primary tumor size, extra-adrenal tumor location, infiltration of peri-adrenal tissue, and the presence of germline pathogenic variants of the succinate dehydrogenase subunit B (*SDHB*) gene.^2,6–8^ In fact, up to 50% of MPPGLs are associated with *SDHB* pathogenic variants.^8,9^

Patients with MPPGL exhibit decreased overall survival compared to patients with no metastases, with only 60% of patients alive 5 years after initial diagnosis.^10^ Nevertheless, MPPGLs are heterogeneous in nature.^11,12^ Some patients have MPPGLs that progress rapidly and are associated with very poor prognosis. Conversely, other patients have MPPGLs that, for unknown reasons, exhibit minimal or no growth over time and are asymptomatic. These patients may achieve a normal lifespan and might not need systemic therapy.^10^ However, most MPPGLs exhibit slow to moderate progression over time, which indicates systemic therapy.

The oldest and most prescribed systemic therapy for patients with progressive MPPGL worldwide is cyclophosphamide, vincristine, and dacarbazine (the CVD protocol). This protocol has never been evaluated prospectively in patients with MPPGL. A meta-analysis of retrospective studies of CVD for MPPGL suggested that 37% of patients might benefit from this treatment.^13^ Although some reports suggest that *SDHB* variant carriers respond better to CVD, these studies are considerably limited by their retrospective nature and lack of a comparison group.^14^ In the author’s experience, some cases respond to CVD while some do not. In addition, CVD is associated with frequent serious adverse events, such as bone marrow insufficiency, neuropathy, gastrointestinal dysmotility, and catecholamine crisis, which might limit the effectiveness of CVD.^15^ Thus, there is a critical need to identify other active or effective treatments for patients with MPPGL.

MPPGLs express various receptors that can be targeted by radiopharmaceuticals.^16^ For example, more than 90% of MPPGLs express receptors for somatostatin in the cell membrane. Patients with MPPGL might therefore benefit from evaluation with functional studies such as gallium-68 DOTATATE positron emission tomography (PET).^17^ This nuclear medicine imaging modality might identify bone metastases that are not obvious in conventional radiographic studies, multifocal lesions in the context of hereditary disease, peritumoral lymphadenopathies for surgical planning, and potential therapeutic targets.^17^ One such radiopharmaceutical target is the noradrenaline transporter, which about 50%–60% of MPPGLs express in their cell membranes.^18^ Patients with MPPGL might also benefit from evaluation with the I-123-meta-iodobenzylguanidine (MIBG) scan. Although this test is not as sensitive as gallium-68 DOTATATE PET and the rate of false-negative results is high,^19^ the test helps identify patients who are candidates for I-131-MIBG therapy.^20^

**Figure 1.**
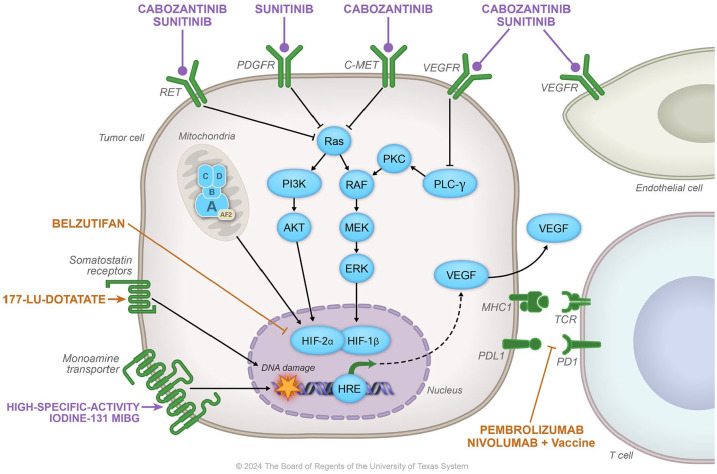
The figure describes the mechanism of action of the therapies described in this manuscript. Therapies that are active and/or effective against MPPGL are highlighted in lavender. Therapies currently evaluated in trials are highlighted in orange. MPPGL, metastatic pheochromocytomas and paraganglioma.

Although therapeutic options are limited, substantial progress has been made in the recognition of effective systemic therapies for these tumors. This review will discuss the results of recent trials in MPPGL with radiopharmaceuticals and tyrosine kinase inhibitors and their impact on current clinical practices, along with guidance in trial design for testing novel agents in MPPGL. The trials selected for discussion have demonstrated that the medications are active and/or effective, with results that represent clinically meaningful benefits. These trials are registered in ClinicalTrials.gov and have published results. Figure 1 describes the mechanisms of action of these therapies.

## Genomics

PPGLs are currently classified into three clusters depending on their molecular phenotypes.^21^ Cluster 1 is the pseudohypoxia cluster; these tumors are characterized by the activation of pathways that mimic the hypoxia signaling of either the Krebs cycle or the hypoxia-signaling pathway. Disruption of the Krebs cycle results in the impairment of mitochondrial oxidation and the accumulation of metabolites such as succinate and fumarate, with the subsequent stabilization of hypoxia-inducible factor-alpha (HIF-α) and promotion of abnormal cell growth and angiogenesis.^21^ These tumors might originate in parasympathetic and/or sympathetic autonomic nervous system ganglia, including the adrenal medulla. When hormonally active, they secrete excessive amounts of dopamine and noradrenaline, but never adrenaline. Most MPPGLs described in clinical practice belong to cluster 1.

Cluster 2 PPGLs are characterized by tyrosine kinase abnormalities that lead to tumor growth through activation of the PI3K/AKT, RAS/RAF/ERK, and mTORC1/p70S6K signaling pathways.^21^ These tumors mainly originate in the adrenal medulla (pheochromocytomas) and rarely in the sympathetic paraganglia (sympathetic paragangliomas).^22^ In the reports that describe sympathetic paragangliomas, it is not clear whether these tumors originated in adrenal medulla remnants or represent tumor seeding after initial surgery for a pheochromocytoma.^23^ Cluster 2 pheochromocytomas are characterized by an excessive secretion of adrenaline and noradrenaline. A small number of MPPGLs described in clinical practice belong to cluster 2 (<10%).

Finally, cluster 3 PPGLs are characterized by an overactive Wnt/beta-catenin signaling pathway.^21^ These tumors were recently described and seem to be rare, and subsequently, their clinical phenotype is currently unknown; however, the rate of metastases associated with these tumors is apparently elevated.

Germline or somatic driver pathogenic variants have been recognized in two-thirds of PPGLs. Approximately 30%–40% of PPGLs are associated with germline pathogenic variants, making PPGL the tumor type most commonly linked to hereditary predisposition in oncological endocrine practice. Subsequently, genetic counseling and testing are recommended for every patient with PPGL. Moreover, approximately 20%–30% of apparently sporadic PPGLs (lacking germline pathogenic variants) are associated with somatic pathogenic variants. Germline pathogenic variants of *VHL*, the succinate dehydrogenase complex (*SDHA*, *SDHB*, *SDHC*, and *SDHD*) and its cofactor *SDHAF2*, fumarate hydratase (*FH*), malate dehydrogenase (*MDH2*), *GOT2*, *SLC25A11*, *EGLN1*, and *EGLN2* genes and somatic mutations of *VHL* and *EPAS1* (*HIF2A*) are associated with the cluster 1 PPGL phenotype. Germline pathogenic variants of *RET*, *NF1*, *TMEM127*, and *MAX* genes, and somatic pathogenic variants of the *BRAF*, RAS family, and *FGFR1* genes are associated with a cluster 2 PPGL phenotype. Last, cluster 3 PPGLs are associated with somatic *CSDE1* pathogenic variants and *MAML3* fusion genes.

## Radiopharmaceuticals

### Conventional MIBG

Wieland et al.^24^ first described the MIBG molecule in 1979. A small molecule, MIBG is an analog of noradrenaline that targets the noradrenaline transporter. MIBG is iodinated in the meta position, which makes the molecule highly resistant to in vivo metabolism. In fact, when a solution of MIBG is infused into an individual, approximately 90% of the medication is eliminated intact in urine, and 40%–50% of this elimination happens over a period no longer than 24 h.^25^ Of interest, MIBG can be labeled with iodine-131 (I-131) instead of non-radioactive iodine. Once I-131-MIBG bonds with the noradrenaline receptor, the molecule is transported inside the MPPGL cells, releasing lethal radioactivity.^25^ A similar effect was previously found in patients with differentiated thyroid cancer.^26^ Thus, I-131-MIBG was introduced as a potential treatment for MPPGLs in 1983.^27^ Initial clinical observations suggested that some patients benefited from I-131-MIBG at doses of 200–500 mCi.^28–32^ However, for more than 20 years, there was no prospective evidence obtained through a clinical trial that could confirm that I-131-MIBG was an active, effective, and safe treatment for patients with MPPGL. Although the medication was prescribed in the United States and several other countries around the world as a treatment for MPPGL, its prescription was rather arbitrary regarding indications, doses, and frequency.

It was not until 2009 that a clinical trial of I-131-MIBG was developed and published for the first time.^33^ This was the first ever published clinical trial for patients with MPPGL treated with any type of systemic therapy and included 50 patients with MPPGL. It is unclear whether these patients had disease progression, and quality of life was not evaluated. Patients were treated with very high doses of I-131-MIBG that varied from 800 to 1200 mCi per infusion. The provided dose was chosen arbitrarily, as no phase I clinical trial was previously developed. The high doses were chosen because a substantial number of MPPGLs exhibit a large tumor burden. The clinical trial suggested that I-131-MIBG was associated with positive signaling, with some patients achieving partial responses (PRs), as demonstrated by follow-up radiographic studies (objective response rate (ORR) = 22%). However, the clinical trial failed to demonstrate that the high-dose I-131-MIBG was safe. The toxicity was overwhelming, and several patients experienced grade 3–5 adverse events. The toxicity profile included severe hypertension during or immediately after the infusion of I-131-MIBG, including catecholamine crisis (20%). Also seen was severe bone marrow insufficiency, which in some patients, unfortunately, did not respond to supportive measures such as transfusion of red blood cells, platelets, and/or granulocyte colony-stimulating factors, leading to the need for stem cell transplant; these patients’ long-term outcomes were not reported. Furthermore, a few patients rapidly developed lethal bone marrow dysplasia. For the first time, pulmonary complications were described in patients treated with I-131-MIBG; a few patients developed irreversible and lethal bronchiolitis obliterans organizing pneumonia and acute respiratory distress syndrome.^33^ The scientific community was concerned by these results; in the United States, the prescription of I-131-MIBG for patients with MPPGL declined, and the treatment of MPPGL mainly focused on chemotherapy with CVD.

### Clinical trials of high-specific-activity MIBG

The beginning of the 21st century brought the development of high specific activity (HSA) I-131-MIBG. This medication is different from the conventional MIBG, as it is produced by a much more refined process through a resin called Ultratrace.^34^ As a result, all molecules in the HSA-I-131-MIBG solution are radioactive, and the dose of radiation delivered to the tumor per dose is much higher than that of conventional MIBG (92.5 vs 1.59 MBq/µg).^25^ The latter is now referred to as low specific activity (LSA) I-131-MIBG.^25^ HSA-I-131-MIBG was only available in the United States.

With the advent of this new option, the US Food and Drug Administration (FDA) mandated a phase I clinical trial of HSA-I-131-MIBG. This was the first phase I clinical trial of systemic therapy specifically devoted to patients with MPPGL.^35^ The trial revealed positive signaling against MPPGL, including PRs and better blood pressure control, and more importantly, the trial identified the maximum tolerated dose (MTD) of HSA-I-131-MIBG. All patients underwent dosimetry. The MTD was 500 mCi (8 mCi/kg/dose for patients smaller than 62.5 kg) given twice every 3 months (with a maximum total dose of 1000 mCi).^35^

Subsequently, a phase II clinical trial of HSA-I-131-MIBG was developed for patients with MPPGL who had hypertension due to excessive secretion of catecholamines.^20^ Begun in 2009, the trial included 68 patients recruited by 7 institutions in the United States. The trial demonstrated that HSA-I-131-MIBG was active and safe for patients with MPPGL. The primary endpoint, mandated by the FDA, was blood pressure control, achieved when patients discontinued or lowered the dose and/or number of their antihypertensive medications by at least 50% compared with their baseline for at least 6 months. From a retrospective perspective, although the primary endpoint was important, as it addressed a measurable clinical primary endpoint, its definition was arbitrary, making its interpretation somewhat challenging. In the trial, 25% of patients achieved the primary endpoint and all patients exhibited some degree of tumor size reduction, including PRs.^20^ Of interest, most patients (~90%) who did not reach the primary endpoint also demonstrated tumor size reduction that was confirmed with follow-up imaging studies.^20^ All these patients exhibited stable and easier-to-manage hypertension compared with their baseline. In fact, most of these patients lowered the dose and/or discontinued antihypertensives but did not fulfill the definition of the primary endpoint.^20^ ORR was an important secondary endpoint for evaluating the antitumor actions of HSA-I-131-MIBG. In the trial, the disease control rate was 92%, and the ORR was 24%.^20^ Although most patients had stable disease (SD), the majority of this group exhibited some degree of tumor size reduction (Table 1).

**Table 1.** Summary of the outcomes of successful clinical trials and real-world studies with cabozantinib, sunitinib, and HSA-I-131-MIBG.

Pharmacokinetic/Pharmacodynamic features, Clinical responses, and Correlations	Cabozantinib^36^	Sunitinib^37,38^	HSA-I-131-MIBG^20,39,40^
Therapeutic target	VEGFR2, RET, c-MET	VEGFR1–3, PDGFR-β, RET	Monoamine transporter
Dose	60 mg daily with dose titration down to 40–20 mg daily	37.5 mg daily	8 mCi/kg/dose or 500 mCi every 3 months × 2 cycles Dosimetry is required.
Administration	Oral	Oral	Intravenous
Speed of action	Fast	Fast	Slow and progressive
Types of positive radiographic responses (RECIST 1.1)	PR, SD	PR, SD	CR (rare), PR, SD
Median PFS	16.6 months	8.9 months	Not reached Mean PFS 36.7 ± 5.7 months
Biochemical correlations	No	Unknown	Yes
Blood pressure control in hormonally active MPPGL	Yes (initially hypertension followed by blood pressure improvement over time)	Expected, not reported	Yes
Main adverse effects	Hypertension and cardiovascular disease	Hypertension and cardiovascular disease	Bone marrow insufficiency
Genotypes associated with positive responses	Sporadic *SDHB* *RET* *VHL*	Sporadic *SDHB* *RET* *VHL*	Sporadic *SDHB* *SDHC* *RET* *VHL*

CR, complete response; HSA, high specific activity; MIBG, meta-iodobenzylguanidine; MPPGL, metastatic pheochromocytoma and paraganglioma; PFS, progression-free survival; PR, partial response; RECIST, Response Evaluation Criteria in Solid Tumors; SD, stable disease; VEGFR2, vascular endothelial growth factor receptor 2.

This trial demonstrated for the first time that radiopharmaceuticals work slowly and progressively over time.^20^ Three months after treatment, the ORR was only 6% of patients, but this number progressively increased to 24% by 1 year after treatment.^20^ The patients also showed improvement in the concentrations of biomarkers.^39^ Patients had MPPGL that mostly secreted noradrenaline. Most patients demonstrated either normalization or a reduction of more than 50% in the concentrations of plasma and urinary noradrenaline, plasma and urinary fractionated normetanephrines, and chromogranin A over time.^39^ Three months after treatment, 21% of patients showed normalization or reduction of normetanephrines by more than 50% compared with their baseline; this percentage progressively increased to 44% by 1 year after treatment.^39^ More importantly, the improvements in these biomarkers significantly correlated with improvements in blood pressure and the oncological responses of tumor size reduction and stabilization, with the most impressive responses becoming more obvious 1 year after the last treatment.^39^

Additional results presented in 2022 also indicated that the clinical, oncological, and biochemical responses to HSA-I-131-MIBG correlated with significant improvements in quality of life, evidenced by functional scales of cognitive, emotional, physical, and social functioning and by symptoms scales related to appetite, constipation, fatigue, insomnia, and pain.^41^ Of interest, at baseline and during treatment, most patients did not describe the classical symptoms of catecholamine excess, such as palpitations, headaches, or diaphoresis^41^; this aligns well with the authors’ experience. Also of interest, patients described a substantial improvement in financial concerns; many were able to return to or continue working, and the expenses related to their medical care declined as their health improved.^41^ So far, these observations represent the most compelling evidence of how a systemic therapy could impact the quality of life of patients with MPPGL.

However, HSA-I-131-MIBG is associated with adverse events. In the phase II trial, the most common and concerning adverse effect was bone marrow suppression.^20^ Approximately 60% of patients treated with HSA-I-131-MIBG developed some degree of bone marrow insufficiency 4–12 weeks after treatment, most commonly affecting platelets. In 23% of patients, bone marrow insufficiency was categorized as severe, requiring supportive measures such as transfusion of platelets and/or red blood cells, and/or granulocyte colony-stimulating factors. No patients required stem cell transplants or died because of bone marrow insufficiency, and all affected patients recovered from this adverse effect^20^ (Table 1). Concerns about leukemia or bone marrow dysplasia development have been raised. Subsequently, the FDA mandated a 10-year phase IV trial to evaluate the long-term benefits and adverse effects of HSA-I-131-MIBG in the patients treated in the phase II trial. This phase IV trial is ongoing.

Of note, patients did not develop catecholamine crisis and/or severe hypertension during or immediately after the infusion of HSA-I-131-MIBG.^20^ This is a notably positive quality of HSA-I-131-MIBG and likely related to the purity of its composition, which prevents the medication from antagonizing and subsequently raising the concentrations of circulating catecholamines around their receptors.^25^ To date, this is the only medication for patients with MPPLG that is not associated with this endocrine complication. HSA-I-131-MIBG became the first FDA-approved therapy for patients with MPPGL.

The phase II clinical trial of HSA-I-131-MIBG did not provide correlations with germline genetic mutations as genetic discrimination, especially by health insurance companies, was a real concern. The trial was designed and approved in 2007, before the adoption of Title II of the Genetic Information Nondiscrimination Act, which prohibits genetic discrimination and took effect in the United States on November 21, 2009.

### Real-world study and clinical availability of HSA MIBG

The only real-world study to date of HSA-I-131-MIBG after its FDA approval was recently published.^40^ Real-world studies help to monitor and evaluate the safety of approved drugs and support the effectiveness of therapy. This study included 25 patients treated with off-label HSA-I-131-MIBG. The study showed that the noradrenaline transporter could be expressed in tumors of different germline genotypes, including patients with paraganglioma syndrome types 3 and 4, von Hippel–Lindau (VHL) disease, multiple endocrine neoplasia type 2 (MEN2), and apparently sporadic tumors. For the first time, the study demonstrated that some patients with MPPGL achieved complete responses. Of note, these responses were seen in patients with paraganglioma syndrome type 4 (*SDHB*).^40^ In addition, impressive PRs were detected in patients with VHL disease, MEN2, and several apparently sporadic tumors.^40^ The ORR was 38% and the disease control rate was 83%.^40^ These favorable tumor responses were associated with improvement in catecholamine concentrations compared with baseline, and 70% of patients discontinued antihypertensives.^40^ Many of these patients later achieved durable therapeutic responses. To date, at least 24% of patients have responded to HSA-I-131-MIBG for longer than 3 years with no additional intervention. The longest recorded duration of response is 6 years and continues.^40^ As this study did not follow the inclusion/exclusion criteria of the phase II clinical trial, HSA-I-131-MIBG was also administered to several patients with mild to moderate bone marrow dysfunction. Bone marrow dysfunction in patients with MPPGL is in fact a common scenario in clinical practice. As described above, bone metastases are prevalent in patients with MPPGL; these lesions displace the bone marrow and frequently require radiation therapy.^4^ Furthermore, patients with MPPGL are frequently treated with CVD chemotherapy or lutetium DOTATATE, and these treatments also might lead to bone marrow insufficiency. Some patients with grade 1 and 2 bone marrow insufficiency received the first dose of HSA-I-131-MIBG, and ~15% of patients developed grade 3 or 4 bone marrow insufficiency, but the majority tolerated the treatment well^40^ and the ones with severe bone marrow dysfunction recovered with supportive measures (Table 1).

In concordance with the phase II clinical trial, this real-world study did not show catecholamine crisis or severe hypertension to be associated with HSA-I-131-MIBG.^40^ Conversely, a few weeks after the treatment was given, several patients noticed improvement in previously high blood pressure because of symptoms of hypotension. Indeed, these symptoms were the first indication of a good therapeutic response to HSA-I-131-MIBG.^40^ This real-world study also showed an adverse effect that was not previously described: a young woman developed premature menopause.^40^ This observation leads us to believe that fertility counseling should be offered to any woman at a fertile age treated with a high-dose radiopharmaceutical medication.^8^

Of note, the study included patients with non-hormonally active tumors and normal blood pressure who also benefited from HSA-I-131-MIBG. One of these patients had a metastatic head and neck paraganglioma. Only 20% of head and neck paragangliomas express the noradrenaline transporter^42^ and might benefit from I-131-MIBG.

Finally, some patients exhibited durable benefits with a single dose of HSA-I-131-MIBG. This approach may be associated with a lower risk of long-term bone marrow adverse effects^40^ and should be considered when prescribing MIBG therapy. A short-term follow-up on the patient’s symptoms and quality of life, with radiographic and biochemical studies, might help clinicians determine if a second dose is needed.

Unfortunately, HSA-I-131-MIBG was discontinued from clinical practice by its manufacturer in 2024 because of its expensive cost and lack of demand. The latter was due in part to reports expressing concerns about the treatment’s activity and safety in older patients, *SDHB* carriers, patients with bone metastases, patients treated with prior systemic therapies, and/or patients with tumors larger than 3 cm in comparison with other radiopharmaceuticals, although these others are not FDA approved and lack prospective evidence derived from clinical trials devoted to MPPGL.^43^ Hence, in the author’s experience, many patients with large tumors, bone lesions, old age, and/or prior systemic therapies were not treated with HSA-I-131-MIBG, even though the phase II clinical trial included patients with these characteristics, who benefited from this treatment.^20,44^ In addition, several patients with paraganglioma syndrome type 4 were not evaluated for HSA-I-131-MIBG because some literature indicated that *SDHB*-positive tumors do not express the noradrenaline transporter.^45,46^ Like patients with apparently sporadic MPPGL, patients with MPPGL associated with SDHX pathogenic variants might or might not express the noradrenaline transporter as well.^40^ However, given current limits in understanding, there remains the potential for patients with MPPGL to benefit from these therapies regardless of genotype, and only evidence derived from functional nuclear medicine studies can determine whether a patient will benefit from any systemic therapy with radiopharmaceuticals.^40^

It is unclear whether HSA-I-131-MIBG will again become available in clinical practice. Nevertheless, the knowledge learned from its trials and real-world study applies to the development of future clinical trials with systemic therapies for MPPGL, including radiopharmaceuticals such as lutetium DOTATATE or the novel alpha emitters Pb-212-DOTAMTATE (AlphaMedix Orano Med, Plano, Texas) and meta-astatobenzylguanidine ([211At]MABG), which have shown promising responses in patients with tumors that express the somatostatin receptors or the noradrenaline transporter, respectively.^47,48^ Moreover, in most countries around the world, the management of MPPGL currently still relies on systemic chemotherapy with CVD or conventional/LSA-I-131-MIBG.^49–51^ The scientific information derived from the trials and real-world studies with HSA-I-131-MIBG may guide clinicians in the use of LSA-I-131-MIBG to treat MPPGL.

## Tyrosine kinase inhibitors

As MPPGLs are vascular tumors, they can be targeted by antiangiogenic medications that also have antiproliferative actions.^52^

### Sunitinib

Sunitinib is a tyrosine kinase inhibitor that inhibits the vascular endothelial growth factor (VEGF) receptors 1–3, the platelet-derived growth factor receptors, and the RET receptor. The first randomized, double-blind, placebo-controlled clinical trial for patients with progressive and unresectable MPPGL was recently published (FIRSTMAPP).^37^ The study included 78 patients with MPPGL who were randomized 1:1 to sunitinib versus placebo. Patients were treated with sunitinib by mouth (37.5 mg daily). The primary endpoint was progression-free survival (PFS) at 12 months. Conventional imaging studies were performed every 3 months. Patients treated with placebo who exhibited disease progression were transitioned to the sunitinib branch; patients treated with sunitinib who exhibited disease progression discontinued participation in the trial. The trial met the primary endpoint. At 1 year, the median PFS rate was 39% in patients treated with sunitinib (90% CI 23%–50%) and 19% in those treated with placebo (90% CI: 11%–31%).^37^ Patients treated with sunitinib exhibited a significantly longer median PFS duration of 8.9 months (95% CI: 5.5–12.7 months) compared with the placebo group (median 3.6 months, 95% CI 3.1–6.1 months). The ORR was 36% and the disease control rate was 72%; for patients with pathogenic variants of the *SDHB* gene, the ORR was 50%. There were no complete responses.^37^ The adverse event profile was similar to that described in other trials with sunitinib.^53^ Hypertension was an adverse event of special interest. Thirteen percent of patients experienced severe hypertension; however, this percentage was not statistically significantly different from that of the placebo group. Overall, this trial demonstrated that sunitinib is effective for patients with MPPGL.^37^ The authors did not describe whether the responses to sunitinib correlated with biomarkers. Information provided by the trial on quality of life was limited but positive. While PFS significantly differed between the groups, the overall survival in patients treated with sunitinib was not significantly different from that seen with the placebo, likely because of the crossover design.^37^ The trial also showed that invariably, cases of MPPGL will develop resistance to sunitinib. Nonetheless, this trial provides the highest level of evidence of effectiveness and safety related to any systemic therapy for MPPGL. Sunitinib is also expected to show activity in MPPGLs associated with other pathogenic variants such as *RET* and *VHL*^38,54^ (Table 1).

### Cabozantinib

Cabozantinib is a potent antiangiogenic medication. Cabozantinib inhibits the VEGF receptor 2, the RET receptor, and the c-MET receptor.^52^ The inhibition of the c-MET receptor is of particular interest, as the c-MET pathway is involved in the distant spread of tumors as well as the development of resistance to medications.^55^ Cabozantinib has shown positive actions in the bone microenvironment that lead to better outcomes in patients with bone metastases^56^; as discussed before, MPPGLs frequently spread to the skeleton.

A single-arm phase II clinical trial of cabozantinib for 17 patients with progressive and unresectable MPPGL (the Natalie Trial) was recently published.^36^ Patients were treated with cabozantinib at 60 mg by mouth daily; the dose was titrated down to 40 or 20 mg daily depending on the severity of adverse events and patients’ response to supportive measures. Patients were evaluated with conventional imaging studies every 2 months. The clinical trial met the primary endpoint of ORR. The ORR was 25% and the clinical benefit rate was 93%; of note, the ORR for patients with pathogenic variants of the *SDHB* gene was 60%.^36^ Almost all patients with SD had some degree of tumor regression; most of these patients had large tumors that, although substantially decreased in size, did not reach PR status (e.g., a patient with target lesions that measured 10 cm at baseline and decreased by 2 cm after cabozantinib was considered to have SD per RECIST 1.1). Radiographic responses were noticed rapidly once the treatment with cabozantinib was started. The PFS duration was 16.6 months (95% CI: 8.1–34.9 months), and the PFS rate at 12 months was 58.8% (95% CI: 39.5%–87.6%).^36^

Although hypertension is a common adverse event related to cabozantinib,^57^ no patients had grade 4 or 5 hypertension. Only one patient exhibited grade 3 hypertension; this patient discontinued cabozantinib for a few days while the antihypertensive medications were adjusted to keep normal blood pressure. This patient restarted cabozantinib at a dose of 40 mg daily and responded for longer than 2 years. All other patients exhibited grade 1 or 2 hypertension upon treatment initiation and required adjustment of the antihypertensive doses without modifying the dose of cabozantinib. Nevertheless, in most patients, the hypertension improved over time. For unclear reasons, radiographic responses did not correlate with biomarkers; antihypertensives might have falsely raised the levels of plasma metanephrines in some cases. Although 82% of patients had bone metastases, treatment with antiresorptive therapy was not provided, and no skeletal-related events were reported during the clinical trial.

The Natalie Trial evaluated samples for pathogenic mutations and amplifications of 136 genes associated with cancer development, including more than 90% of driver genes associated with MPPGL (described in the “Genomics” section of this manuscript). Most samples were primary tumors that were previously removed. Surprisingly, none of these tumors had pathogenic variants except for the cases with germline pathogenic variants of *SDHB* (30%) that have been identified by next-generation sequencing as part of standard-of-care genetic testing offered to every patient with MPPGL.^36^ The trial did not find somatic pathogenic variants of the c-MET gene or amplification of its pathway, suggesting that the actions of cabozantinib mainly derive from its antiangiogenic properties.^36^

The study had limitations, as there were very few samples derived from metastases. A biopsy is contraindicated in patients with MPPGL, as it may predispose them to a catecholamine crisis; liquid biopsies may become an option to overcome this obstacle.^58^ In addition, the Natalie Trial did not evaluate for pathogenic variants of *EPAS1* or genes associated with cluster 3 PPGL. Like the FIRSTMAPP trial, the Natalie Trial indicated that patients develop resistance to cabozantinib at variable times.^36^ Cabozantinib is predicted to offer benefits to patients with MPPGL associated with different genotypes^59^ (Table 1).

## Ongoing clinical trials

Promising clinical trials for patients with MPPGL are ongoing, providing medications with different mechanisms of action from those described above. All these trials are registered at ClinicalTrials.gov. Figure 1 describes the mechanism of action of the therapies evaluated by these clinical trials.

### Belzutifan

The HIF-2α inhibitor belzutifan is currently being evaluated through an international, single-arm phase II clinical trial for patients with unresectable and progressive MPPGL. This medication targets the “heart” of the cluster 1 MPPGLs.^60,61^ The clinical trial, however, allows the participation of patients with MPPGL irrespective of their cluster/genotype, and comparisons between clusters might be possible. The primary endpoint of the trial is ORR. The treatment with belzutifan is offered at a dose of 120 mg daily (NCT04924075). Belzutifan is FDA approved for the treatment of VHL-related kidney cancer, pancreatic neuroendocrine tumors, hemangioblastomas that do not need surgery right away, and advanced kidney cancer that has been previously treated with immunotherapy and antiangiogenic therapies such as cabozantinib or sunitinib.^62,63^ The MTD of belzutifan has not been determined. In the trials for patients with kidney cancer and VHL-related tumors, belzutifan exhibited an impressive safety profile. Belzutifan was associated with occasional severe adverse events (mainly anemia and hypoxia) that responded well to supportive therapies.

### Lutetium-177 DOTATATE

Lutetium-177 DOTATATE targets the somatostatin receptors, releasing lethal radiation to the tumor cell. These receptors modulate hormone secretion, induce apoptosis, and inhibit cell proliferation. The medication is approved by the regulatory agencies of various countries including the FDA for the treatment of patients with gastroenteropancreatic neuroendocrine tumors.^64^ The dose is 200 mCi intravenously every 2 months for 4 cycles. More than 100 publications on this drug describe a positive signal for the treatment of MPPGL. However, these studies to date have been retrospective and have many limitations on clear clinical applicability.^65,66^ These studies frequently indicate that the medication is effective and safe, potentially leading to overly liberal use; however, only prospective studies can prove that a medication is either active or effective and safe. Although most publications indicate that patients tolerate this treatment well, recent reports and the authors’ experience indicate that patients with hormonally active MPPGL can develop severe catecholamine crisis/hypertension during and/or immediately after the infusion, and admission to the ICU might be required.^67^ In the author’s opinion, this is an expensive medication that sometimes leads to disease stabilization for some time, and occasionally to impressive radiographic responses. Patients must be well treated with antihypertensives in preparation for treatment.

A phase II clinical trial for patients with MPPGL is ongoing. The primary endpoint is PFS at 6 months, and the study compares *SDHB*-positive MPPGL with apparently sporadic tumors. The study excludes cluster 2 MPPGL (NCT03206060).

### Lanreotide

Lanreotide is an effective medication for patients with gastro-entero-pancreatic neuroendocrine tumors with Ki67 < 10%. The CLARINET trial demonstrated that patients treated with lanreotide achieved a significantly longer PFS when compared with placebo and its toxicity was quite acceptable.^68^ Given the high expression of somatostatin receptors in MPPGL, the NCCN guidelines recommend the use of the somatostatin analogs lanreotide and octreotide to stabilize the disease and to improve hormonal symptoms including hypertension.^69^ However, the evidence to support this recommendation is scant. In fact, two small clinical trials failed to demonstrate the activity of octreotide for MPPGL. In these trials, the hormonal symptoms, blood pressure, and biomarkers did not improve.^70,71^ A prospective clinical trial is evaluating the activity of lanreotide in MPPGL (LAMPARA). The primary endpoint is tumor growth rate in comparison with pre-enrollment growth rates (NCT03946527).

### Checkpoint inhibitors

The role of immunotherapy for patients with MPPGL remains to be determined. A phase II clinical trial with pembrolizumab devoted to patients with MPPGL has been developed (NCT02721732). The study demonstrated a low ORR of 9%, PFS of 5.7 months (95% CI: 4.37–not reached), and median survival duration of 19 months (95% CI: 9.9–not reached) with positive responses independent of patients’ hereditary backgrounds, hormonal status, and the presence of infiltrating mononuclear inflammatory cells or PD-L1 expression.^72^ The most impressive response was noticed in a patient previously exposed to infection by *Pseudomonas aeruginosa*, raising new questions about the mechanisms responsible for positive responses to immunotherapy.^73^ The SPENCER trial is now evaluating the checkpoint inhibitor nivolumab in combination with EO2401, a vaccine that provides three microbiome-derived CD8+ epitopes mimicking parts of MPPGL-associated antigens. The endpoints are safety, immunogenicity, and preliminary efficacy (NCT04187404).

### Olaparib (PARP inhibitor) and temozolomide (alkylating agent)

Poly (adenosine diphosphate-ribose) polymerases (PARPs) are proteins that help repair deoxyribonucleic acid mutations. PARPs can be activated by the accumulation of succinate in MPPGL associated with *SDHB* pathogenic variants and hence represent a potential target. Case reports indicate that MPPGL associated with pathogenic *SDHB* variants might respond to temozolomide,^74^ although the responses might be independent of the tumor genotype.^75^ Altogether, the addition of olaparib to temozolomide might lead to disease stabilization and prevent the emergence of tumor resistance. A phase II clinical trial is comparing temozolomide versus temozolomide plus olaparib, with a primary endpoint of PFS (NCT04394858).

## Lessons that can be applied to future trial design

Although some concepts learned from clinical trials with systemic therapies for other cancers—including other neuroendocrine tumors—could be extrapolated to MPPGL, we must remember that MPPGL are unique tumors. Hypertension due to catecholamine excess is a real concern; treatment with antihypertensives before and during trial participation must be strict, and the therapeutic dosages of tyrosine kinase inhibitors and other therapies must be individualized and chosen carefully. Tyrosine kinase inhibitors at high doses may lead to severe hypertension and/or catecholamine crisis due to their direct toxicity toward the patient’s vasculature and the excessive secretion of catecholamines due to tumor destruction.^76^ Illustrating this, previous phase II trials with lenvatinib and pazopanib for MPPGL prescribed high doses (lenvatinib 20 mg/daily) and/or titrated the doses up (pazopanib was titrated from 400 to 800 mg daily), making it difficult for patients to tolerate these treatments.^77,78^ Some patients discontinued treatment and trial recruitment failed. Conversely, the FIRSTMAPP trial prescribed sunitinib at a lower dose (37.5 mg/daily) than what has been recommended for patients with kidney cancer (50 mg/daily), and the NATALIE trial prescribed cabozantinib at an intermediate dose of 60 mg daily and titrated the dose down; these dose modifications likely made it possible for patients to tolerate these treatments well.^36,37^

Bone metastases are very common in patients with MPPGL, up to 20% of whom only have bone metastases.^4,79^ However, these patients are frequently excluded from clinical trials because, for the most part, these lesions are not measurable. Clinical trials should consider enrolling these patients as an exploratory group where functional imaging modalities such as FDG-PET, gallium-68 DOTATATE PET, and/or MIBG scans could be used to evaluate therapeutic responses. The NATALIE trial is the first trial exploring patients with bone metastases only. Patients were followed with an FDG-PET scan.^36^ Results are pending for publication. In fact, functional imaging modalities should be evaluated as exploratory endpoints in patients with measurable disease as well. This approach will help us understand how these imaging modalities can help evaluate therapeutic responses and complement the information derived from conventional studies such as CT and MRI. A proposal for MIBG scan evaluation has been recently published.^80^ Gallium-68 DOTATATE PET standardized uptake value (SUV) variations have not been prospectively studied in patients with MPPGL treated with systemic therapy. SUV variations might not correlate with tumor response to therapy; in this order of ideas, a composite endpoint is important to explore. A composite endpoint might include the development of new lesions, an increase in biomarkers such as plasma metanephrines, a lack of blood pressure control, and/or the development of skeletal-related events.

Molecular analysis of primary tumors and metastases is ideal, providing important clues for identifying effective and personalized therapies as well as mechanisms of resistance. Biopsies are relatively contraindicated in patients with hormonally active tumors, as they may be predisposed to catecholamine crisis; however, samples for molecular analysis might be obtained in other ways. As patients with MPPGL frequently have large primary tumors, their resection might prevent local complications, improve hormonal manifestations and overall survival, and facilitate responses to systemic therapy^81^—while providing samples for molecular analysis. The NATALIE trial evaluated samples from primary tumors that were removed time before the trial. As discussed before, no somatic pathogenic variants were identified.^36^ Alternatively, liquid biopsies are not invasive procedures and are easier and faster to obtain than regular biopsies. Although still exploratory, liquid biopsies of MPPGL may provide us with important molecular information at different stages of treatment.^58^ The belzutifan trial is exploring this option.

The duration of response of patients treated with tyrosine kinase inhibitors is limited. There is a need to identify other therapies that combined with these medications could provide more durable responses. A sequential combination of tyrosine kinase inhibitors for patients with clear cell renal cell carcinoma treated with cabozantinib after pazopanib or sunitinib has been demonstrated to be effective.^82,83^ The Natalie Trial described the systemic therapies provided to patients with MPPGL after discontinuation of cabozantinib.^36^ No patients were treated with sunitinib; however, two patients were treated with lenvatinib as the first subsequent therapy. Although these patients did not apparently benefit from lenvatinib, the question remains unanswered. Therefore, clinical trials for patients with MPPGL should explore sequential therapies. It is not clear what the cut point for sensitivity might be; however, PFS at 6 months could be worthwhile to try with a second tyrosine kinase inhibitor given the limited number of therapies.

In the same order of ideas, the concomitant combination of tyrosine kinase inhibitors, radiopharmaceuticals, and immunotherapy must be explored in clinical trials; although checkpoint inhibitors alone do not seem to be very active for patients with MPPGL,^72^ the concomitant use of radiopharmaceuticals and/or tyrosine kinase inhibitors with immunotherapy might enhance the immune response.^73,84,85^ However, to evaluate the combination of therapies is very expensive and the rarity of MPPGL could limit the interest in developing this type of trial. Basket trials that include other tumors such as gastro-entero-pancreatic neuroendocrine neoplasms—in addition to MPPGL—could be a valuable option to demonstrate either therapeutic activity or effectiveness.

The clinical trials mentioned here were developed in high-income countries. Given the well-known genetic diversity observed in PPGL and its potential to guide a desired “personalized medical care,” more data from across the globe are needed. Clinical trials must include a diverse population from all continents. Table 2 describes the current limitations of studying systemic therapies in clinical trials and their potential solutions.

**Table 2.** Current limitations and potential solutions to study systemic therapies for patients with MPPGL in clinical trials.

Domain	Current limitations	Future directions
Precision therapy		
Genomics	Lack of information regarding the distribution of PPGL clusters in distinct areas around the world	Development of clinical and genomic databases through international consortiums to obtain a better understanding of genetic and ethnic variability
Individualized therapy	Biopsy of MPPGL to determine the best possible targeted therapy may be harmful to individuals with functional tumors	Liquid biopsy
Interpretation of clinical benefit	Some trials have included patients with stable disease	Multicentric international clinical trials including only patients with progressive MPPGL
Sequential therapy	Randomized clinical trials are difficult to perform due to the disease’s rarity	Aim for global inclusivity in clinical trials and real-life studies under diverse local realities
Assessment of responses in patients with bone metastases	20% of MPPGL cases present with bone metastases only	Symptoms and skeletal-related events should be considered as endpoints in addition to cross-sectional imaging and PET assessment
Systemic treatment/theranostics		
Conventional MIBG therapy	Lack of prospective clinical trials	Better-designed clinical trials with different dosing strategies
	Arbitrary dosing and treatment protocols	Monitoring for long-term benefits and side effects; Phase I trial development
	High doses are associated with severe toxicities including bone marrow insufficiency, pulmonary complications, and fatal outcomes	Consider better patient selection, lower doses, and single-dosing strategies; Dosimetry-based prospective studies
High specific activity MIBG	Concerns about long-term adverse effects, including potential leukemia	Phase IV trials to monitor long-term outcomes
	Recent discontinuation from clinical practice	Uncertainty about reintroduction; Application of the knowledge derived from this therapy in the design of future clinical trials with other systemic therapies
Tyrosine kinase inhibitors	Emergence of resistance to sunitinib and cabozantinib	Investigation of therapeutic combinations to enhance efficacy and reduce resistance
	Frequent adverse events, particularly hypertension	Explore personalized dosing strategies based on patient tolerance, ethnicity, and genetics
Immunotherapy	Low overall response rates in clinical trials	Further research into biomarkers to identify potential responders; Combined therapy with tyrosine kinase inhibitors and/or radiopharmaceuticals
	Limited understanding of the immunological responses in MPPGL	Combination of checkpoint inhibitors with vaccines or other immunotherapy approaches to enhance response
Lutetium-177 DOTATATE	Limited clinical data regarding efficacy and safety	Prospective multicentric clinical trials
Novel radiopharmaceuticals	Large tumor heterogeneity in terms of potential targets for radioligand therapy	Investigation of novel agents such as Pb-212-DOTAMTATE for efficacy in MPPGL; Combination/alternating radiopharmaceutical therapy

MIBG, meta-iodobenzylguanidine; MPPGL, metastatic pheochromocytomas and paraganglioma; PET, positron emission tomography.

## Conclusion

I-131-MIBG and radiopharmaceuticals alike work slowly and progressively over time and are therefore recommended to patients with MPPGL who do not exhibit rapid progression. Although I-131-MIBG may occasionally lead to complete response or remission, PR or SD is more often the result, with SD being the most common radiographic response. Durable responses lasting longer than 5 years are possible. In fact, a durable SD response might represent a very good outcome despite minimal or no tumor size reduction. Radiographic and blood pressure responses to I-131-MIBG correlate with biomarkers and hence serve as surrogate markers of response. I-131-MIBG can be offered to any hereditary genotype if the tumor expresses the noradrenaline transporter. Bone marrow insufficiency is the most concerning adverse event associated with I-131-MIBG. However, treatment with I-131-MIBG in patients with mild to moderate baseline bone marrow deficiencies is feasible, if no safer therapeutic options exist.

Cabozantinib and sunitinib work fast, with radiographic responses observed shortly after the start of treatment. CRs have not been described, PR happens often, and SD, usually with some degree of tumor reduction, is the most common clinical outcome. Durable responses lasting longer than 3 years are rare. Although radiographic responses do not correlate with biomarkers, in patients with PR or SD with tumor size reduction, their blood pressure might improve over time. Cabozantinib and sunitinib might be offered to any hereditary genotype. Cardiovascular toxicity due to hypertension is the most concerning adverse event associated with these therapies. However, with adequate blood pressure care, these patients may not be at any major risk of hormone-related symptoms at treatment initiation. Conversely, their blood pressure might improve over time.

Knowledge of a patient’s genetic profile can aid in selecting the proper therapy and regimen. The results of the published and successful clinical trials for patients with MPPGL show that the indication for therapy with radiopharmaceuticals or tyrosine kinase inhibitors cannot be determined yet by the tumor genotype and that treatment should be provided depending on clinical features such as the speed of disease progression and/or the expression of receptors for radiopharmaceuticals. Of the ongoing clinical trials, the phase II trial with Belzutifan might provide us with the first evidence of a personalized therapy for MPPGL.

## Declarations

**Ethics approval and consent to participate:** Not applicable.

**Consent for publication:** Not applicable.

**Availability of data and materials:** Not applicable.
